# Neonatal tetanus in undocumented migrant families in Saudi Arabia: outcomes and critical care resource utilization in a 10-year tertiary PICU cohort

**DOI:** 10.3389/fped.2026.1807654

**Published:** 2026-08-19

**Authors:** Samah Al-Harbi, Abeer A. Alnajjar, Fidaa Al Maghrabi, Fatin A. Basnawi, Khouloud A. Al-Sofyani, Mohammed S. Uddin

**Affiliations:** 1 Department of Pediatrics, Faculty of Medicine, King Abdulaziz University, Jeddah, Saudi Arabia; 2 Department of Pediatrics, King Abdulaziz University Hospital, Jeddah, Saudi Arabia; 3 Pediatric Infectious Disease Unit, King Abdulaziz University Hospital, Jeddah, Saudi Arabia; 4 Pediatric Intensive Care Unit, King Abdulaziz University Hospital, Jeddah, Saudi Arabia; 5 Clinical Skills and Simulation Center, Faculty of Medicine, King Abdulaziz University, Jeddah, Saudi Arabia; 6 Department of Pediatrics, Ministry of National Guard Health Affairs, Dammam, Saudi Arabia; 7 King Abdullah International Medical Research Center, Riyadh, Saudi Arabia; 8 King Saud bin Abdulaziz University for Health Sciences, Riyadh, Saudi Arabia

**Keywords:** emigration and immigration, intensive care units, pediatrics, length of stay, mechanical ventilation, Saudi Arabia, tetanus, neonatal

## Abstract

**Background:**

Neonatal tetanus (NT) is an entirely preventable disease; however, it persists among underserved populations even within high-resource health systems. Contemporary data detailing outcomes in referral pediatric intensive care units (PICUs)—specifically regarding the intersection of low mortality and substantial resource utilization—remain limited.

**Methods:**

We conducted a retrospective cohort study of 74 neonates with clinically confirmed NT admitted to a tertiary academic PICU in Saudi Arabia between 2014 and 2023. The cases met World Health Organization clinical criteria. We evaluated admission severity (Ablett grade), presenting clinical features and baseline laboratory indices as prognostic factors. Notably, postadmission therapies were excluded to preserve baseline prognostic interpretation and minimize confounding by indication. In-hospital mortality was analyzed using Firth bias-reduced logistic regression. Furthermore, resource-utilization outcomes—including invasive mechanical ventilation (IMV) duration, PICU length of stay (LOS), and hospital LOS—were modeled using accelerated failure time (AFT) regression. Internal validation for parsimonious mortality models utilized bootstrap optimism correction.

**Results:**

In-hospital mortality was 4.1% (3/74), occurring exclusively among infants graded Ablett III at admission. Conversely, resource utilization was substantial: median (IQR) IMV duration was 25.0 (16.0–30.0) days, PICU LOS was 32.0 (20.0–37.0) days, and hospital LOS was 40.0 (27.3–57.5) days. In parsimonious admission-only Firth models, higher birth weight showed an inverse association with lower mortality [adjusted odds ratios (aOR) 0.35 per SD, 95% confidence interval (CI) 0.09–0.85], while admission C-reactive protein showed a risk-increasing trend (aOR 1.81 per SD, 95% CI 0.87–3.92); given the fact that there were only three deaths, these estimates should be interpreted as directional signals rather than definitive independent predictors. In AFT models, septic shock on admission was associated with a shorter observed IMV duration (time ratio 0.05, 95% CI 0.002–0.947), most consistent with a mortality-related truncation of observed duration rather than accelerated clinical recovery. At 3–6 months, neurodevelopmental follow-up was available for 71 survivors, of whom 10 screened positive for developmental concerns (10/71; 14.1%).

**Conclusion:**

In this tertiary PICU cohort of neonatal tetanus in Saudi Arabia, mortality was low but critical-care utilization and early morbidity were substantial. Admission severity and inflammatory burden showed higher-risk signals, whereas birth weight showed a protective association; however, the small number of deaths limits precise inference. These findings quantify the PICU burden of a preventable disease and reinforce prevention priorities, including maternal vaccination, antenatal care, and safe delivery practices.

## Background

Neonatal tetanus (NT) is entirely preventable; however, when it occurs, it typically presents as a high-intensity pediatric critical illness characterized by prolonged ventilatory dependence, extended ICU length of stay (LOS), and complication-prone trajectories that consume substantial pediatric intensive care unit (PICU) resources. In a Durban PICU cohort, NT admissions required sustained critical-care exposure, including prolonged invasive ventilation and extended ICU admission, underscoring the burden of the system among survivors (1). Consistent with this systems-level perspective, an ICU evaluation from Tanzania reported that protocolized tetanus management increased the intensity of supportive interventions without a commensurate reduction in mortality (2). Notably, sepsis accounted for an increasing proportion of deaths over time, aligning with the clinical reality that prolonged invasive support can shift risk toward infection-related morbidity and mortality (2). Accordingly, NT should be viewed not only as a vaccine-preventable infection but also as a condition that imposes a sustained critical-care burden among survivors.

At the population level, the global control framework—maternal and neonatal tetanus elimination (MNTE)—is deliberately district-focused, defining elimination as fewer than one NT case per 1,000 live births per year in every district (3). Nevertheless, sustaining elimination requires continuous delivery of tetanus toxoid–containing vaccination and clean birth practices, supported by district risk analysis and surveillance designed to detect residual transmission in underserved groups (4). WHO guidance also emphasizes that NT is often under-recognized in routine systems (a “silent” outcome in communities with limited access to care), meaning that national-level rarity can mask localized vulnerability until clusters appear clinically (4, 5). Therefore, contemporary tertiary-center cohorts remain valuable for characterizing clinical trajectories and resource utilization patterns that surveillance systems may fail to capture.

Even in high-resource health systems where NT is uncommon, preventable risk can persist in socially marginalized populations, particularly among migrant families with disrupted preventive care and incomplete vaccination histories. A US sentinel case involving an infant born to immigrant parents highlighted missed prevention opportunities during prenatal care, illustrating how routine processes can fail to identify and mitigate residual risk (6). More broadly, evidence syntheses indicate that migrants and refugees can experience undervaccination driven by structural barriers such as fragmented access, documentation gaps, and discontinuity of care (7). Likewise, serologic data demonstrate that these barriers translate into clinically meaningful susceptibility among newly arrived migrants lacking vaccination documentation (8). Consequently, “rare disease” epidemiology at the national level may coexist with concentrated vulnerability in specific subgroups.

Within Saudi Arabia, NT continues to be reported predominantly in contexts characterized by home delivery, inconsistent antenatal follow-up, and inadequate maternal immunization—patterns consistent with residual risk concentrated outside routine facility–based obstetric care (9, 10). These local observations align with MNTE principles: when clean delivery and maternal tetanus protection are not equitably achieved for all groups, neonatal tetanus persists as a predictable consequence of access and coverage gaps.

Contemporary critical-care literature further emphasizes that severe tetanus commonly entails weeks of organ support, with outcomes shaped not only by spasm control but also by autonomic instability and secondary complications during prolonged invasive care (11). ICU cohort data also show high rates of nosocomial infections (including ventilator associated pneumonia) and early mortality clustering in the most severe cases, supporting the interpretation that early deterioration can “truncate” duration-based outcomes rather than reflect accelerated recovery (12). Practical ICU management reviews likewise highlight dysautonomia and infection risk as dominant threats once airway control and sedation are established, reinforcing the need to interpret resource-use outcomes in the context of competing risks and prolonged exposure to hospital-acquired complications (13). In parallel, surveillance evaluations demonstrate substantial underascertainment of NT in routine reporting in some settings, particularly when deaths occur outside facilities, thereby obscuring “pockets of risk” (14).

Despite the recognized severity of NT in intensive care, important knowledge gaps remain regarding contemporary burden in tertiary PICUs serving undocumented/underserved migrant populations—particularly the quantification of critical-care resource utilization [duration of invasive mechanical ventilation (IMV), PICU length of stay, and total hospital length of stay] and early survivorship morbidity (1, 2, 5, 11–13). Accordingly, we conducted a 10-year retrospective cohort study (2014–2023) of WHO-defined neonatal tetanus in a tertiary academic pediatric ICU in Saudi Arabia serving a large undocumented migrant population, with the aim of quantifying in-hospital mortality and critical-care resource utilization, evaluating admission severity, presenting clinical features and baseline laboratory indices as exploratory prognostic signals, and assessing early morbidity among survivors using standardized developmental screening at 3–6 months.

## Methods

### Study design and setting

We conducted a retrospective cohort study of neonates with clinically confirmed NT admitted to the PICU of a tertiary academic hospital in Saudi Arabia between 1 January 2014 and 31 December 2023. The PICU serves as a regional referral center and provides care for a substantial number of families without regularized residency status. Interfacility transfers were eligible. Length-of stay outcomes were calculated from the time of admission to our PICU (and index hospital encounter) to PICU/hospital discharge, transfer out, or death.

### Definition of undocumented migrant status

Undocumented migrant status was operationalized using administrative registration fields in the electronic medical record. Patients were classified as undocumented when a valid national identification or residency number was absent and this classification was supported, when available, by non-national/uninsured registration category, charity-care pathway, or social-work documentation. Ambiguous cases were independently reviewed by two investigators and resolved by consensus.

### Data sources and collection

Data were extracted from the EMR using a structured abstraction template that captured demographics, maternal/perinatal history, presenting features, severity measures, laboratory and radiologic findings, complications, and clinical outcomes through discharge and follow-up. Source documents included clinician and nursing notes, medication administration records, laboratory and microbiology reports, radiology reports, and discharge summaries. Follow-up information (3–6 months postdischarge) was abstracted from outpatient clinic records. Before analysis, the dataset underwent internal consistency checks (range checks and cross-field validation of dates, procedures, and therapies). Discrepancies were resolved by a re-review of the underlying source documentation.

### Case definition, eligibility, and severity classification

Case ascertainment followed World Health Organization surveillance standards for neonatal tetanus. A suspected case was defined as a neonate who fed and cried normally during the first 2 days of life and then, between days 3 and 28, either developed a tetanus-compatible illness or died. A confirmed case met all three clinical criteria: (i) normal sucking and crying during the first 2 days of life; (ii) inability to suck normally between days 3 and 28; and (iii) subsequent stiffness and/or spasms. We included confirmed NT after a chart review and excluded presentations with a more plausible alternative diagnosis.

We defined the incubation period as the interval from birth to the first tetanus symptom. The period of onset was defined as the interval from the first symptom (e.g., difficulty sucking or trismus) to the first generalized spasm; shorter intervals were considered markers of greater clinical severity. We excluded non-neonatal tetanus (>28 days of age), presentations lacking the characteristic sequence (loss of sucking followed by rigidity/spasms), and cases in which an alternative diagnosis was more consistent with the clinical course (e.g., meningitis or metabolic disorders).

Severity at PICU admission was graded using the Ablett classification. Grade I denotes mild to moderate trismus with generalized spasticity without spasms or respiratory compromise. Grade II indicates marked rigidity with brief spasms and mild to moderate respiratory compromise. Grade III reflects severe trismus and generalized spasticity with prolonged spasms, apneic spells, and severe dysphagia. Grade IV includes grade III features plus autonomic dysfunction, such as labile hypertension or tachycardia alternating with hypotension or bradycardia. Autonomic features were prespecified and abstracted from clinical documentation across the PICU stay.

### Outcomes

The primary outcome was all-cause in-hospital mortality, defined as death occurring before discharge from the PICU or the index hospitalization, ascertained from EMR documentation and death registry fields. We also reported PICU mortality (death before PICU discharge). Secondary outcomes captured resource utilization and included IMV duration, PICU LOS, and hospital LOS. IMV duration was measured from the first endotracheal intubation to successful liberation from IMV for ≥48 consecutive hours; reintubation within 48 h was considered part of the same IMV episode. Tracheostomy day was considered the end of the acute IMV episode. PICU and hospital LOS were calculated from admission to discharge; for patients who died, LOS was measured to the date of death. Interfacility transfers were recorded, and LOS to transfer was reported.

### Complications and clinical definitions

Complications were prespecified and adjudicated retrospectively using standard surveillance definitions. Ventilator-associated pneumonia (VAP) was defined using CDC/NHSN pediatric PNEU criteria. Central line–associated bloodstream infection (CLABSI) followed CDC/NHSN Patient Safety Component definitions, with central-line days recorded and attribution rules applied according to NHSN guidance. Sepsis and septic shock were defined according to the Surviving Sepsis Campaign pediatric guidelines; septic shock at presentation required vasoactive support and/or persistent hypoperfusion documented within the first hours of PICU admission.

### Neurodevelopmental follow-up

Neurodevelopmental status at 3–6 months postdischarge was assessed using standardized tools used in the follow-up clinic. An abnormal Ages and Stages Questionnaire, Third Edition (ASQ-3) screen was defined as any domain score below the age-specific cutoff or within the monitoring zone requiring referral. When the Bayley Scales of Infant and Toddler Development, Fourth Edition (Bayley-4) was administered, corrected composite scores (mean 100, SD 15) were reported, and values <85 were classified as delayed. The assessment instrument used, assessor discipline, and follow-up completeness were recorded. Follow-up outcomes were summarized descriptively and restricted to survivors with available assessments.

### Ethical considerations

The study was approved by the Local Research Ethics Committee (REC), Unit of Biomedical Ethics, King Abdulaziz University Hospital (KAUH) (NCBE Registration No. HA-02-J-008), as a non-interventional retrospective cohort study (Reference No. 433-25). The requirement for informed consent was waived because of the retrospective design. All extracted data were deidentified and managed in accordance with the Declaration of Helsinki.

### Statistical analysis plan

Baseline characteristics were reported as counts (%) for categorical variables and medians (IQR) for continuous variables. Between-group comparisons used Fisher's exact test for categorical variables and the Mann–Whitney *U*-test for continuous variables, as appropriate. All tests were two-sided with a significance threshold of *α* = 0.05. The primary inferential framework comprised (i) Firth bias-reduced logistic regression for in-hospital mortality and (ii) accelerated failure-time (AFT) models for resource utilization. Prespecified robustness checks for rare events and exploratory machine-learning screening were conducted and presented explicitly as sensitivity or hypothesis-generating analyses. Given the very small number of deaths, mortality models were intended to identify admission-only prognostic signals rather than definitive independent predictors. Firth bias-reduced logistic regression was used to reduce small-sample bias and mitigate separation; however, all effect estimates were interpreted cautiously, with emphasis on direction, magnitude, and uncertainty rather than statistical significance alone.

### Primary outcome (in-hospital mortality)

Because of the very small number of deaths, associations with in-hospital mortality were estimated using Firth bias-reduced logistic regression to reduce small-sample bias and mitigate separation. Bedside severity at PICU admission was prespecified as the main exposure (Ablett grade III + vs. lower/unknown). To limit confounding by indication, postadmission therapies were not included in prognostic models. Two parsimonious admission-only models were fitted: (Model 1) Ablett III + and birth weight (*z*-standardized, per 1 SD) and (Model 2) Ablett III + and C-reactive protein (CRP; z-standardized, per 1 SD). The results were presented as adjusted odds ratios (aORs) with profile-likelihood 95% confidence intervals and penalized likelihood-ratio *p* values. Model discrimination was summarized using the area under the receiver operating characteristic curve (AUC). Calibration was described using the calibration intercept (calibration-in-the-large) and calibration slope, supported by calibration plots. Internal validation used bootstrap optimism correction (*B* = 1,000 resamples). These metrics are reported to characterize in-sample model behavior, with interpretation guided by the rare-event context. Baseline laboratory indices were defined as the first recorded values at/near PICU admission (prior to escalation of definitive tetanus-directed ICU therapies when feasible) to preserve prognostic temporality.

### Modified Dakar score (mDakar3)

A three-item modified Dakar score (mDakar3) was constructed from admission fever (yes/no), spasms/convulsions (yes/no), and period of onset <48 h (yes/no), assigning one point per component (range 0–3). Because of extreme class imbalance, discrimination was explored using class-weighted ridge logistic regression with death as the outcome and mDakar3 as the sole predictor. We reported the odds ratio per one-point increase, bootstrap confidence intervals (*B* = 1,000), and the apparent AUC.

### Secondary outcomes

IMV duration, PICU LOS, and hospital LOS were modeled using AFT regression. Log-normal and Weibull distributions were compared, and the best-fitting form was selected using the Akaike information criterion. Effects are reported as time ratios (TR), where TR > 1 indicates a longer observed duration and TR < 1 indicates a shorter observed duration. Because death competes with time-to-liberation and discharge endpoints, AFT estimates were interpreted as associations with observed durations; in particular, shorter observed times in higher-severity strata may reflect truncation by early death rather than accelerated recovery. To further evaluate this possibility, we performed a descriptive competing-risk sensitivity analysis using cumulative incidence functions, treating death as a competing event for IMV liberation, PICU discharge, and hospital discharge (Supplementary Figure S3). Given the very small number of deaths (*n* = 3), this analysis was intended to support interpretation of duration-based outcomes rather than to provide formal competing-risk regression estimates. Accordingly, Fine–Gray and cause-specific hazard models were not fitted because such models would be statistically unstable in this rare-event setting. As a robustness check, negative-binomial regression was applied when overdispersion was evident; AFT models were considered the primary analyses.

### Sensitivity and exploratory analyses

Prespecified sensitivity analyses included exact (conditional) logistic regression for key binary predictors, rare events ridge logistic regression to stabilize estimation under collinearity, and Bayesian logistic regression with weakly informative priors, reporting posterior median odds ratios with approximately 95% credible intervals. As a clearly labeled hypothesis-generating analysis, a composite severe outcome (death OR septic shock OR IMV ≥14 days OR VAP/CLABSI) was evaluated using class-weighted elastic-net logistic regression with stability selection. Only admission covariates were included, and continuous predictors were *z*-standardized. For this exploratory screen only, minimal single imputation was used for baseline covariates; the results were reported as selection frequency and directional stability rather than effect estimates.

### Missing data

Missingness was quantified for all candidate predictors prior to modeling. Primary inferential models (Firth mortality models and AFT resource-utilization models) used complete-case analysis. For the exploratory machine-learning screen only, baseline covariates were singly imputed using a prespecified rule (continuous variables imputed to the cohort median and categorical variables imputed to the modal category); no outcomes were imputed.

### Software, reproducibility, and reporting

Analyses were conducted in *R* and Python. Primary inferential analyses were performed in R using logistf (Firth regression), survival and flexsurv (AFT models), pROC (AUC estimation), and rms (calibration assessment). Exploratory machine-learning analyses were performed in Python using scikit-learn (ridge/elastic-net and resampling). Bootstrap procedures used 1,000 resamples. A fixed random seed was applied for resampling-based procedures (seed = 202401), and package versions are reported in the Supplementary Methods. Reporting followed the STROBE guideline for observational studies**.**

## Results

### Cohort overview and critical care resource utilization

Seventy-four neonates who met World Health Organization (WHO) criteria for neonatal tetanus were included in the analytic cohort (Table 1). Additional cohort descriptors—including nationality distribution, administrative registration category, delivery setting, and cord-care practices—are provided in Supplementary Table S1. Consistent with high-risk perinatal circumstances, all infants had no documented antenatal care and no documented maternal tetanus vaccination; the high prevalence of home delivery and potentially unclean cord-care practices is detailed in Supplementary Table S1 (Table 1). Critical care resource utilization was substantial: among ventilated infants with available ventilation-day data (*n* = 69), median (IQR) IMV duration was 25.0 (16.0–30.0) days; median (IQR) PICU LOS was 32.0 (20.0–37.0) days; and median (IQR) hospital LOS was 40.0 (27.3–57.5) days (Table 1). In-hospital mortality was 4.1% (3/74). Neurodevelopmental follow-up at 3–6 months was available for 71 survivors; 10 of these 71 infants screened positive for developmental concerns (14.1%). These findings are descriptive and limited to infants with available follow-up assessment.

**Table 1:** Baseline characteristics, admission severity, key laboratory measures, and core outcomes (*N* = 74).

Characteristic	Overall (*N* = 74)
Demographics and perinatal factors	
Male sex, *n* (%)	45 (60.8)
Age at symptom onset, days, median (IQR)	8.0 (6.0–10.0)
Birth weight, kg, median (IQR)	3.15 (2.90–3.40)
Maternal age, years, median (IQR)	26.0 (19.0–30.0)
No documented antenatal care, *n* (%)	74 (100.0)
No documented maternal tetanus vaccination, *n* (%)	74 (100.0)
Delivery and cord-care characteristics	
Home delivery, *n* (%)	65 (87.8)
Cord cutting uncleaned, *n* (%)	47 (63.5)
Dirty cord on presentation, *n* (%)	48 (64.9)
Admission clinical features	
Fever, *n* (%)	64 (86.5)
Convulsions/spasms, *n* (%)	31 (41.9)
Septic shock on admission, *n* (%)	70 (94.6)
Admission severity (Ablett grade), *n* (%)	
Ablett I	0 (0.0)
Ablett II	4 (5.4)
Ablett III	70 (94.6)
Ablett IV	0 (0.0)
Key admission laboratory measures	
C-reactive protein (CRP), mg/L, median (IQR)	30.0 (12.2–79.0)
White blood cell count, ×10⁹/L, median (IQR)	16.9 (12.2–20.4)
Platelet count, ×10⁹/L, median (IQR)	301.0 (186.8–431.2)
Core outcomes and resource utilization	
In-hospital mortality, *n* (%)	3 (4.1)
IMV duration, days, median (IQR)^a^	25.0 (16.0–30.0)
PICU length of stay, days, median (IQR)	32.0 (20.0–37.0)
Hospital length of stay, days, median (IQR)	40.0 (27.3–57.5)
Developmental screen positive at 3–6 months among survivors, n/N (%)^b^	10/71 (14.1)

Data are presented as median (IQR) or *n* (%). Percentages use *N* = 74, unless otherwise specified.

CRP, C-reactive protein; IMV, invasive mechanical ventilation; IQR, interquartile range; PICU, pediatric intensive care unit.

a IMV duration was summarized among ventilated infants with available IMV-day data (*n* = 69).

### In-hospital mortality and severity distribution

**Figure 1:**
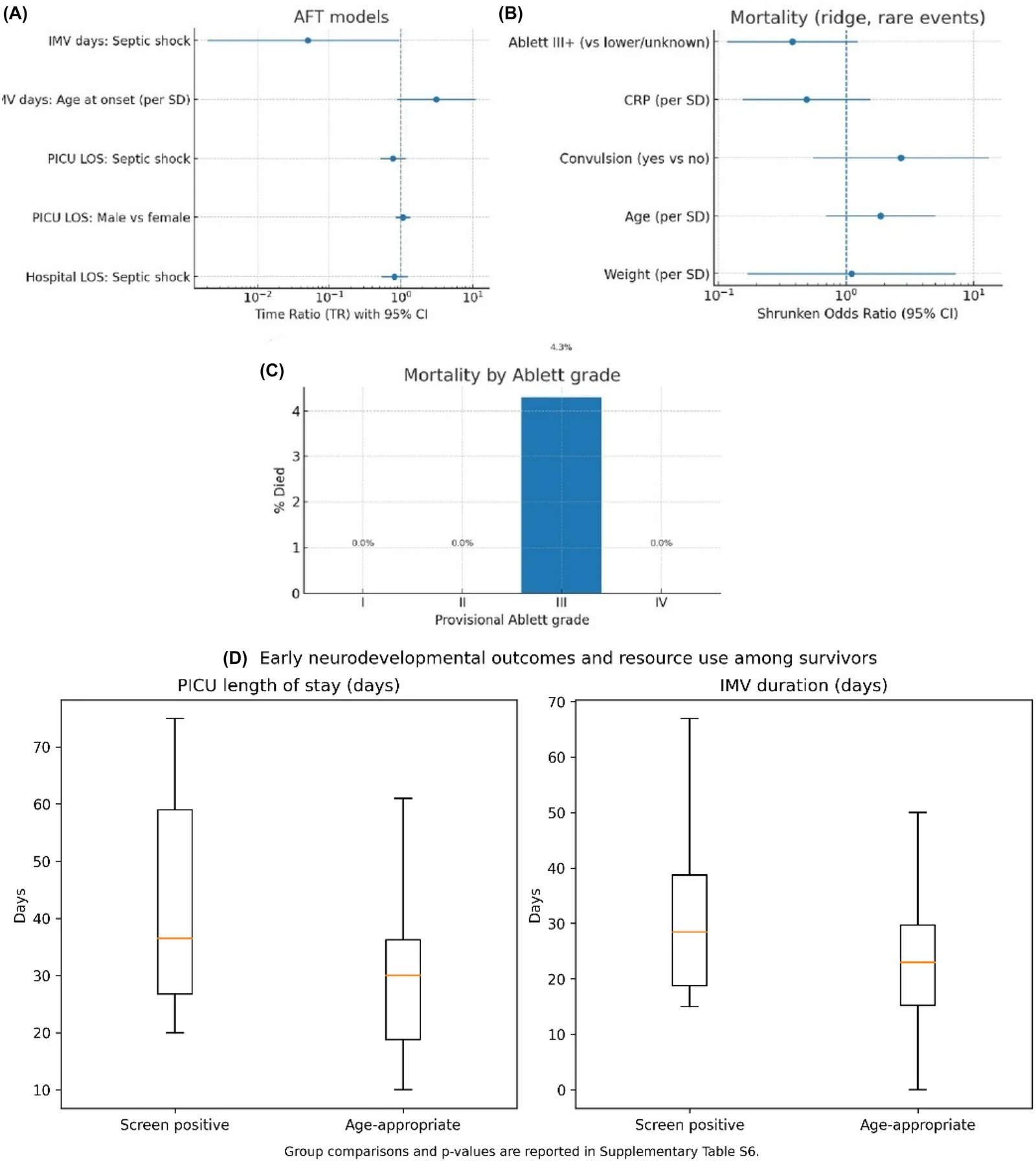
Severity, outcomes, and early neurodevelopment among neonates with tetanus: **(A)** accelerated failure-time (AFT) models for resource utilization, presented as time ratios (TR; log scale) with 95% confidence intervals (CIs) for IMV duration (septic shock; age at symptom onset), PICU length of stay (septic shock; male sex), and hospital length of stay (septic shock). TR > 1 indicates a longer observed duration and TR < 1 indicates a shorter observed duration. Shorter observed durations in higher-risk strata (e.g., septic shock) are interpreted as truncation by early death (observed resource use) rather than accelerated recovery. **(B)** Rare-events ridge logistic regression for in-hospital mortality, presented as shrunken odds ratios (ORs) with 95% CIs for Ablett grade III + (vs. lower/unknown), CRP, convulsions, age at symptom onset, and birth weight. Slopes were penalized (intercept unpenalized), covariates were *z*-standardized, and postadmission therapies were excluded. **(C)** In-hospital mortality by Ablett grade at PICU admission. Deaths occurred only in grade III (3/70; 4.3%); no deaths were observed in grade II (0/4), and no infants were graded I or IV at admission. **(D)** Early neurodevelopmental screening at 3–6 months among survivors. The boxplots show PICU length of stay and IMV duration stratified by screening status (screen positive vs. age-appropriate). Group comparisons and *p*-values are reported in Supplementary Table S6. The dashed vertical reference lines indicate TR = 1 **(A)** or OR = 1 **(B)**. AFT, accelerated failure-time; CI, confidence interval; CRP, C-reactive protein; IMV, invasive mechanical ventilation; LOS, length of stay; OR, odds ratio; PICU, pediatric intensive care unit; TR, time ratio.

In-hospital mortality was 4.1% (3/74) (Table 1). All deaths occurred among infants classified as Ablett grade III at PICU admission (3/70; 4.3%), with no deaths among infants graded Ablett II (0/4) and no infants graded Ablett I or IV at admission (Table 2; Figure 1C). Given the near-uniform admission severity distribution, with most infants classified as Ablett grade III, Ablett grade was not suitable for robust estimation of an independent severity effect. Therefore, severity-grade-specific mortality is presented descriptively, and the Ablett coefficient in regression models should not be interpreted as an independent prognostic estimate. Table 2 is retained to display the complete Ablett distribution and to document the sparse, near-uniform severity structure underlying this descriptive interpretation.

**Table 2:** In-hospital mortality by admission Ablett severity.

Ablett grade	Died (*n*)	Survived (*n*)	Total (*n*)	Mortality (%)
I	0	0	0	—
II	0	4	4	0.0
III	3	67	70	4.3
IV	0	0	0	—
Overall	3	71	74	4.1

Mortality (%) is calculated within each Ablett severity grade as deaths/total  × 100. Empty grade categories are retained to display the complete admission Ablett distribution and to show the near-uniform severity structure of the cohort. “—” indicates not applicable because no infants were graded I or IV at admission.

### Admission-only prognostic signals for in-hospital mortality

Because only three deaths occurred, these associations should be interpreted as exploratory admission-only prognostic signals with substantial uncertainty and not as definitive independent predictors. Mortality associations were evaluated using admission-only Firth bias-reduced logistic regression, with an emphasis on effect direction, magnitude, and uncertainty rather than statistical significance alone (Table 3). In univariable models, higher admission CRP showed a risk-increasing trend (aOR 1.85 per SD), and spasms/convulsions showed a large risk-increasing signal (aOR 10.68), although with imprecise confidence limits. Higher birth weight showed a protective signal for mortality (aOR 0.33 per SD) (Table 3). Other admission features showed no consistent association in this rare-event setting. As a complementary rare-events robustness screen, shrunken odds ratios from ridge logistic regression are displayed in Figure 1B. In prespecified parsimonious multivariable Firth models restricted to admission variables, higher birth weight showed a protective association after accounting for bedside severity (Ablett III+) [Model 1: aOR 0.35 per SD, 95% confidence interval (CI) 0.09–0.85; likelihood-ratio test (LRT) *p* = 0.028] (Table 4). Because birth weight varied within a relatively narrow range in this cohort, the standardized birth-weight estimate should not be interpreted as evidence that small absolute weight differences have a large causal effect on mortality. Rather, in this rare-event analysis, birth weight is best viewed as an exploratory marker of physiologic reserve or unmeasured perinatal vulnerability. In the alternative model substituting CRP for birth weight, higher CRP demonstrated a risk-increasing trend (Model 2: aOR 1.81 per SD, 95% CI 0.87–3.92; *p* = 0.076) (Table 4). Given the near-uniform admission severity (94.6% Ablett III) and only three deaths, the Ablett coefficient is not interpretable as an independent effect and was reported only to reflect the cohort's observed severity distribution (Table 4).

**Table 3:** Univariable baseline associations with in-hospital mortality (Firth bias-reduced logistic regression; admission-only predictors).

Predictor	Adjusted OR (aOR)	95% CI
Ablett grade III + (provisional)	0.47	0.03–7.04
CRP (per SD)	1.85	0.86–3.95
Spasms/convulsions	10.68	0.94–613.25
Birth weight (per SD)	0.33	0.08–0.83
Age at onset (per SD)	0.64	0.16–1.61
Lockjaw/trismus	0.69	0.05–7.54
Fever	0.25	0.02–7.82
CK/CPK (per SD)	0.84	0.02–1.67
Male sex	1.09	0.11–31.91

Odds ratios were estimated using Firth bias-reduced logistic regression because of rare events (three deaths). Continuous predictors were *z*-standardized; odds ratios were expressed per 1 SD increase. All predictors were admission/baseline variables; postadmission therapies were excluded to reduce confounding by indication.

aOR, adjusted odds ratio; CI, confidence interval; CK/CPK, creatine kinase; CRP, C-reactive protein.

**Table 4:** Parsimonious multivariable Firth logistic regression models for in-hospital mortality (baseline-only predictors).

Model	Predictor	Adjusted OR (aOR)	95% CI	*p*-Value (LRT)^a^
Model 1 (Ablett III + + birth weight)	Ablett III + (vs. I–II)	0.35	0.01–5.91	1.000
	Birth weight (per SD)	0.35	0.09–0.85	0.028
Model 2 (Ablett III + + CRP)	Ablett III + (vs. I–II)	0.62	0.04–5.91	1.000
	CRP (per SD)	1.81	0.87–3.92	0.076

Odds ratios were estimated using Firth bias-reduced logistic regression because mortality was rare (three deaths). Continuous predictors were *z*-standardized; odds ratios were reported per 1 SD increase. Postadmission therapies were excluded to reduce confounding by indication. *p*-Values were obtained from penalized likelihood-ratio tests (LRTs). Ablett III + denotes Ablett grade III or IV at presentation; no grade IV was observed. Because admission severity was highly concentrated and events were sparse, the Ablett coefficient was unstable and should not be interpreted as an independent prognostic effect; grade-specific mortality is presented descriptively in Table 2.

aOR, adjusted odds ratio; CI, confidence interval; CRP, C-reactive protein; LRT, likelihood-ratio test.

### Model performance and internal validation

**Figure 2:**
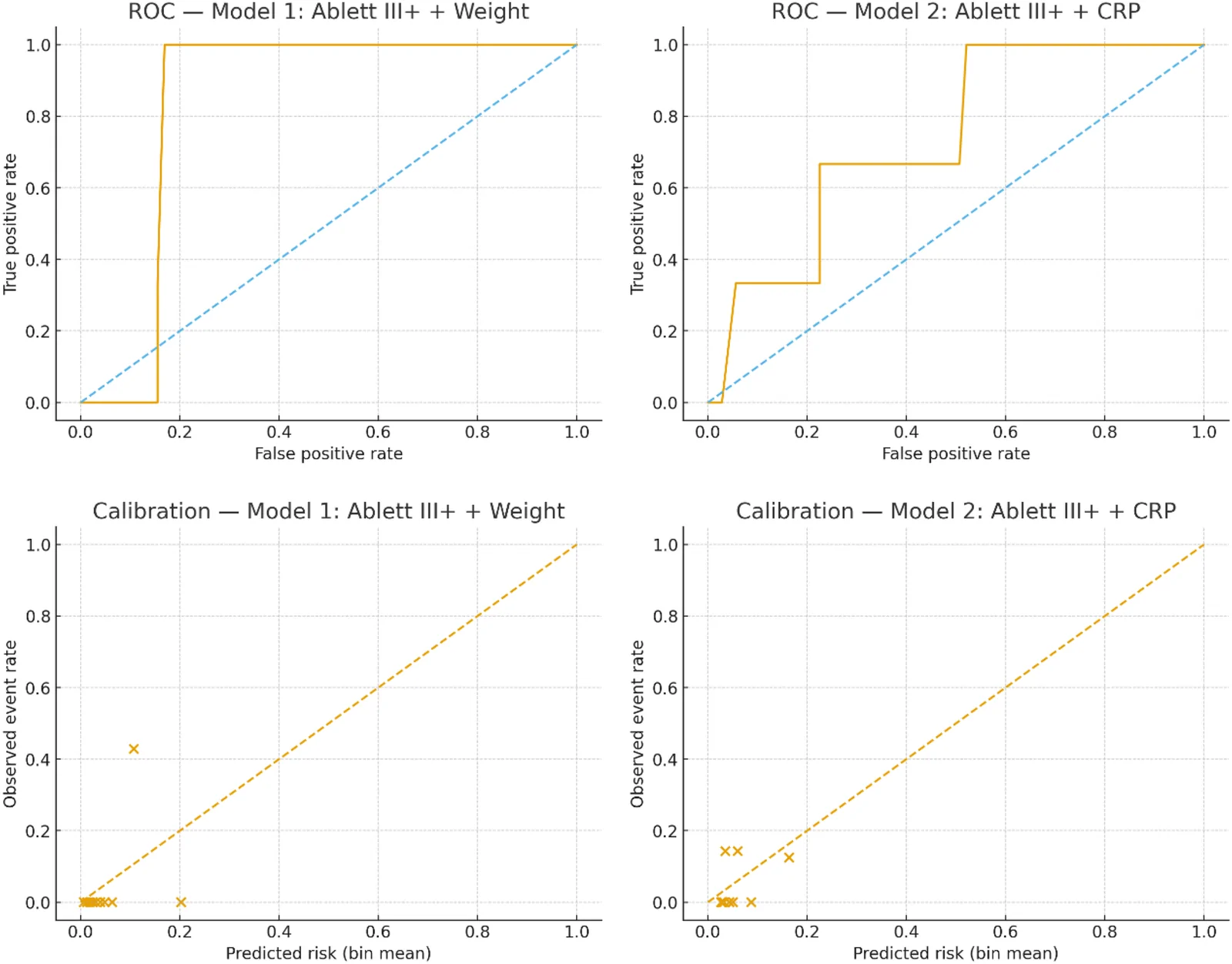
Discrimination and calibration of parsimonious Firth logistic regression mortality models. The top row shows receiver operating characteristic (ROC) curves for (left) Model 1 [Ablett III + plus birth weight (*z*)] and (right) Model 2 [Ablett III + plus admission CRP (*z*)]. The dashed diagonal line indicates no discrimination (AUC = 0.50). The bottom row shows the corresponding calibration plots, comparing observed event rates with mean predicted risk within grouped bins; the dashed diagonal line indicates perfect calibration. The points represent binned estimates; given the rarity of mortality (three deaths), calibration should be interpreted cautiously. Continuous covariates were *z*-standardized, and models used Firth bias-reduced logistic regression with admission-only predictors (postadmission therapies excluded). AUC, area under the receiver operating characteristic curve; CRP, C-reactive protein; ROC, receiver operating characteristic.

Model discrimination was higher for the birth weight model [Model 1: Ablett III + plus birth weight (*z*)] than for the CRP model [Model 2: Ablett III + plus CRP (*z*)] (Table 5; Figure 2). For Model 1, the apparent AUC was 0.84 and remained similar after bootstrap optimism correction (AUC 0.83; *B* = 1,000) (Table 5; Figure 2). For Model 2, discrimination was lower (apparent AUC 0.74; optimism-corrected AUC 0.70) (Table 5; Figure 2). Calibration intercept and slope estimates are summarized in Table 5, with the corresponding calibration plots shown in Figure 2; because of only three deaths, calibration is presented as a descriptive internal check and should be interpreted cautiously (Table 5; Figure 2).

**Table 5:** Discrimination and calibration of parsimonious Firth mortality models.

Model	AUC (apparent)	AUC (optimism-corrected)	Calibration intercept	Calibration slope
Model 1 (Ablett III + + birth weight)	0.84	0.83	−0.36	1.12
Model 2 (Ablett III + + CRP)	0.74	0.70	−0.35	0.95

Apparent performance was calculated in the analytic sample. Optimism-corrected AUC was estimated using bootstrap internal validation (*B* = 1,000 resamples). The calibration intercept reflects calibration-in-the-large (0 indicates no systematic over- or under-prediction). The calibration slope reflects model overfitting (ideal = 1; values <1 suggest overfitting and overly extreme predictions).

AUC, area under the receiver operating characteristic curve; CRP, C-reactive protein.

### Exploratory risk stratification (modified Dakar score (mDakar3)

**Figure 3:**
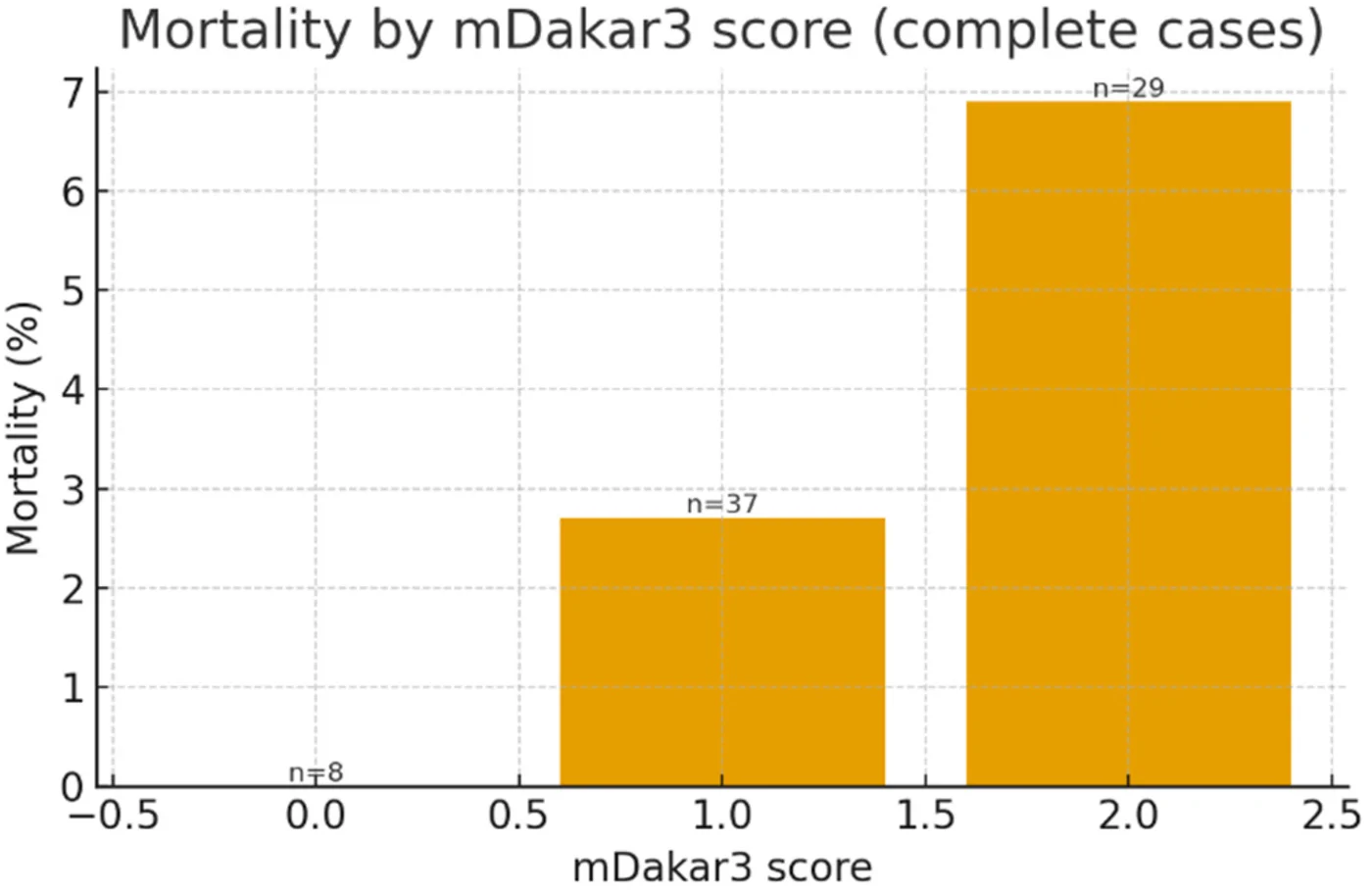
Mortality by modified Dakar score (mDakar3). Bars show in-hospital mortality by score among 74 neonates (3 deaths). mDakar3 = fever + spasms/convulsions + period of onset <48 h (0–3 points). Group sizes: score 0, n=8; score 1, n=37; score 2, n=29; no infant scored 3. Mortality was 0%, 2.7%, and 6.9% for scores 0, 1, and 2, respectively. Estimates are descriptive given only three events.

Modified Dakar (mDakar3) scores were 0 in 8 infants (10.8%), 1 in 37 (50.0%), and 2 in 29 (39.2%). Crude in-hospital mortality increased monotonically across score categories (0%, 2.7%, and 6.9%, respectively). In an exploratory class-weighted ridge screen with mDakar3 as the sole predictor, the odds ratio per 1-point increase was 2.77 (bootstrap 95% CI 0.33–16.51), and discrimination was modest (AUC 0.662; *n* = 74; deaths = 3). Given the small number of deaths, these findings are presented as descriptive and hypothesis-generating (Figure 3).

### Exploratory machine-learning screen for a severe clinical course

To prioritize admission features associated with a more complicated course, we fitted a class-weighted elastic-net model with stability selection for a composite severe outcome (death OR septic shock OR IMV ≥14 days OR VAP/CLABSI), using baseline variables only. Most top predictors were selected in nearly all subsamples; therefore, ranking emphasizes directional stability and coefficient magnitude. Convulsions/spasms showed the most consistent risk-increasing signal, while higher birth weight and older age at symptom onset showed consistently protective directions (Table 6; Supplementary Figure S1).

**Table 6:** Elastic-net stability-selection ranking for a composite severe clinical course (baseline predictors only).

Predictor	Selection frequency^a^	Mean |*β*| (all resamples)	Mean *β* (when selected)	Sign stability^b^
Convulsions	0.99	5.34	5.40	1.00
Birth weight (per SD)	0.99	4.39	−4.43	0.00
Age at onset (per SD)	0.99	3.38	−3.36	0.04
CRP (per SD)	0.99	2.30	1.93	0.78
CK/CPK (per SD)	0.99	2.02	−2.03	0.03
Male sex	0.99	1.84	1.08	0.71
Lockjaw/trismus	0.99	0.84	0.49	0.63

Class-weighted elastic-net logistic regression with stability selection was used to prioritize baseline features, a composite severe clinical course (death or septic shock or IMV ≥14 days or VAP/CLABSI), using admission-only variables (postadmission therapies excluded). Continuous predictors were *z*-standardized prior to modeling.

*β*, regression coefficient; CK/CPK, creatine kinase; CRP, C-reactive protein; IMV, invasive mechanical ventilation; VAP, ventilator-associated pneumonia; CLABSI, central line–associated bloodstream infection.

a *Selection frequency* indicates the proportion of resamples in which the predictor was selected (non-zero coefficient).

b *Sign stability* is the probability that the coefficient was positive among resamples where the predictor was selected (values near 1.00 indicate a consistently positive direction; values near 0 indicate a consistently negative direction).

### Resource utilization (AFT models)

AFT models were used to quantify associations between admission variables and resource utilization outcomes (Table 7; Figure 1A). Septic shock on admission was associated with markedly shorter observed IMV duration (TR 0.05, 95% CI 0.002–0.947; *n* = 69) (Table 7; Figure 1A). In line with the prespecified interpretation framework, this pattern is most consistent with truncation by early death (i.e., reduced observed duration) rather than faster clinical recovery. Directionally similar reductions were observed for PICU length of stay (TR 0.77, 95% CI 0.51–1.16; *n* = 74) and hospital length of stay (TR 0.81, 95% CI 0.53–1.24; *n* = 74), although confidence intervals included 1.0 (Table 7; Figure 1A). Older age at symptom onset tended to be associated with a longer IMV duration (TR 3.08 per SD, 95% CI 0.88–10.85), while male sex showed little association with PICU length of stay (TR 1.06, 95% CI 0.84–1.33) (Table 7; Figure 1A).

**Table 7:** Accelerated failure time (AFT) models for resource-use outcomes.

Outcome	Predictor	Time ratio (TR)	95% CI
IMV duration (days)	Septic shock on admission	0.05	0.002–0.947
IMV duration (days)	Age at onset (per SD)	3.08	0.88–10.85
PICU length of stay (days)	Septic shock on admission	0.77	0.51–1.16
PICU length of stay (days)	Male sex	1.06	0.84–1.33
Hospital length of stay (days)	Septic shock on admission	0.81	0.53–1.24

Accelerated failure-time regression was used to model the duration of invasive mechanical ventilation (IMV), PICU stay, and hospital stay. The error distribution (log-normal or Weibull) was selected using the Akaike information criterion for each outcome. The results were expressed as time ratios (TR) with 95% confidence intervals (CIs); TR > 1 indicates a longer observed duration and TR < 1 indicates a shorter observed duration. Each row represents a separate model with the listed predictor as the covariate; continuous predictors were *z*-standardized (per 1 SD increase). Because death can truncate observed durations, TR < 1 for septic shock is interpreted as truncation by early mortality rather than accelerated recovery. Sample size reflects complete data for each outcome–predictor pair.

### Competing-risk sensitivity analysis

To assess whether death influenced the interpretation of duration-based outcomes, we performed a competing-risk sensitivity analysis using cumulative incidence functions, treating death as a competing event for IMV liberation, PICU discharge, and hospital discharge (Supplementary Figure S3). Across all three endpoints, the cumulative incidence of death remained low throughout follow-up (three deaths; 4.1% overall mortality), whereas most infants ultimately achieved IMV liberation and discharge. These findings support the primary interpretation of the AFT models by demonstrating that shorter observed durations among clinically unstable infants should not be interpreted as faster recovery. Rather, death functioned as a terminal competing event that truncated observed ventilation duration and length-of-stay outcomes.

### Early neurodevelopmental outcomes among survivors

Follow-up status at 3–6 months was ascertained for the cohort. Among survivors (*n* = 71), 14.1% screened positive for developmental concern (10/71), with a binomial 95% CI of 7.8–24.0% (Table 1; Figure 1D). Developmental findings represent early screening outcomes at 3–6 months among survivors with available follow-up and should not be interpreted as definitive long-term neurodevelopmental diagnoses. Compared with age-appropriate survivors, screen-positive infants had a longer PICU length of stay (*p* = 0.035). IMV duration and hospital length of stay were numerically longer in the screen-positive group, but these differences did not reach statistical significance (*p* = 0.245 and *p* = 0.056, respectively) (Figure 1D; Supplementary Table S6). Convulsions/spasms were more frequent among screen-positive infants (*p* = 0.011), and admission CRP tended to be higher (*p* = 0.153) (Supplementary Table S6).

### Sensitivity analyses

Prespecified sensitivity analyses were performed to evaluate robustness under rare events and potential separation, including rare-events ridge models, exact methods, Bayesian logistic regression, and a limited Firth specification incorporating delivery setting. The findings were directionally consistent with the primary interpretation that baseline severity markers—particularly convulsions/spasms and inflammatory burden—tracked higher mortality risk, whereas higher birth weight showed a protective signal, with substantial uncertainty due to only three deaths. Full outputs are provided in Supplementary Tables S2–S5; Bayesian estimates are visualized in Supplementary Figure S2.

## Discussion

### Principal findings: high survival at high cost

Because mortality was rare (three deaths) and admission severity was highly concentrated (94.6% Ablett III), all admission associations were interpreted as exploratory risk signals rather than definitive independent predictors. In this decade-long PICU cohort of undocumented neonates with WHO-defined neonatal tetanus, in-hospital mortality was low (4.1%); however, survivors required prolonged invasive ventilation and extended PICU/hospital stays—an archetypal “high survival at high cost” pattern. Contemporary intensive care (early airway control, targeted sedation/neuromuscular blockade, antimicrobials, antitoxin, and vigilant autonomic/complication management) can reduce the historically extreme case-fatality reported in poorly resourced settings to single digits in specialized units, although at the cost of weeks of organ support and intensive infection prevention measures (15–17). Our time-to-event results add an important benchmarking nuance: septic shock at presentation was associated with a shorter observed invasive ventilation duration and LOS not because of rapid recovery but because early death truncates exposure time—an established analytic hazard in critical-care outcomes when death competes with “time-to-liberation” endpoints (18–21). Mortality clustering in high bedside severity and the parallel signals that inflammation and spasms track risk, while greater physiologic reserve (higher birth weight, older age at onset) is protective are concordant with severity constructs embedded in tetanus grading and prognostic syntheses (11, 16, 22). Importantly, survival did not imply benign recovery: early follow-up identified neurodevelopmental delay among survivors, reinforcing that preventable upstream failures can translate into downstream morbidity even when modern PICU care achieves survival (23–27). These preliminary screening findings suggest potential survivorship morbidity, but a longer standardized follow-up is required to determine persistent neurodevelopmental impairment. Collectively, these data position neonatal tetanus in high-income settings—particularly among marginalized, undocumented families—as a largely preventable but resource-intensive PICU burden: critical care buys survival, whereas durable solutions remain upstream prevention (11–13, 15, 23, 24, 26, 28, 29).

### Admission risk signals and early clinical course

Across complementary approaches—bias-reduced rare-events regression, exact and Bayesian sensitivity checks, and an exploratory class-weighted elastic-net screen—the same signal emerged: greater bedside severity and inflammation tracked with higher early risk, whereas greater physiologic reserve (higher birth weight, older age at onset) appeared protective. In the parsimonious admission-only model, higher birth weight (per SD) showed an inverse association with the odds of in-hospital death, while admission CRP showed a risk-increasing trend; because there were only three deaths, these estimates are best interpreted as directional risk signals rather than definitive independent predictors (19, 20). The birth-weight signal requires cautious interpretation. Although median birth weight was 3.15 kg and the IQR was narrow, the model used a standardized per-SD contrast rather than a per-gram or per-100-g contrast. Therefore, the estimate should not be read as implying that small absolute differences in weight directly caused large differences in mortality. More plausibly, birth weight acted as a global marker of physiologic reserve, perinatal robustness, nutritional reserve, or unmeasured vulnerability at presentation. Given only three deaths, residual confounding and sparse-event instability remain possible; this association should therefore be interpreted as a hypothesis-generating signal rather than a clinically actionable threshold. Two interpretive cautions are essential. First, with only three deaths, uncertainty is unavoidable; therefore, we emphasized direction and cross-method consistency rather than definitive thresholds. Bias-reduction and sensitivity analyses are appropriate for sparse events and mitigate separation and small-sample bias without overstating precision (19, 21, 30, 31). Second, severity markers are partially collinear (e.g., spasms, respiratory compromise, and shock), and limited adjustment can shift point estimates despite stable clinical meaning; accordingly, we prioritized baseline (pretherapy) predictors to reduce confounding by indication (11–13, 15). Clinically, these findings support early admission risk stratification: infants presenting with severe spasms/convulsions, physiologic instability (including shock), and elevated inflammatory markers warrant intensified monitoring for respiratory failure and autonomic crises, aggressive infection prevention, and anticipatory family communication regarding the likelihood of prolonged support (13, 15, 32). Conversely, higher birth weight/older age at onset may indicate a lower early-death risk but does not eliminate substantial morbidity among survivors. Further validation should account for differences in case mix, referral pathways, diagnostic documentation, and treatment protocols before any bedside risk signal is generalized beyond this setting (17, 33, 34).

### Interpreting time outcomes

Why “Shorter” Can Mean Worse: Our time-to-event analyses highlight a central interpretive nuance: in the presence of a terminal event, a shorter observed ventilation duration or LOS does not imply faster recovery. In accelerated failure-time models, septic shock at presentation was associated with a markedly shorter observed invasive ventilation duration and reduced PICU/hospital LOS; given that death truncates exposure, these time ratios reflect earlier mortality rather than therapeutic efficiency. The competing-risk sensitivity analysis reinforced this interpretation. Although septic shock was associated with a shorter observed ventilation duration and length of stay, cumulative incidence functions demonstrated that death acted as a competing event that terminated observation before extubation or discharge could occur (Supplementary Figure S3). Consequently, shorter observed durations in higher-risk infants likely reflected mortality-related truncation rather than accelerated clinical recovery. This is a recognized challenge in ICU outcomes whenever death competes with liberation/discharge and is not explicitly accounted for (20, 21, 30, 35, 36). Practically, time outcomes should be interpreted alongside mortality and, when possible, within frameworks that acknowledge competing risks—cause-specific approaches, subdistribution hazards, and multistate models that represent transitions among ventilation, extubation, discharge, and death (30, 36). Likewise, composite endpoints such as ventilator-free days intentionally blend mortality and duration; however, they can be zero-inflated and may obscure effects or penalize the sickest strata, particularly in small cohorts (20, 21). Accordingly, we recommend that reports of time outcomes in neonatal tetanus pair time ratios with mortality and state explicitly when shorter times are driven by lethality rather than efficiency, to prevent misinterpretation in audits, dashboards, and comparative benchmarking (20, 30, 36).

### Internal validation and exploratory bedside scoring

Our internal validation strategy quantified optimism and clarified transportability while separating confirmatory from exploratory work. Using bootstrap resampling (*B* = 1,000)—preferred for small datasets and prediction models—we observed good discrimination for the parsimonious bedside model and near-ideal optimism-corrected calibration slope, alongside modest calibration-in the-large offset consistent with sparse event learning. Reporting optimism-corrected performance and calibration is standard practice to avoid inflating apparent accuracy; moreover, if externally applied, simple intercept recalibration (and, if needed, uniform shrinkage) should precede routine use (33, 34). Methodologically, bias-reduced regression mitigates separation and finite-sample bias expected with very low event counts, and bootstrap validation avoids data splitting that would further degrade precision (33, 34, 37, 38). Consequently, we interpret the corrected discrimination and calibration slope as proof-of-concept that a minimal bedside model can rank risk robustly; nevertheless, external validation remains a prerequisite for adoption (33, 34).

### Global context: immigrant vulnerability

Neonatal tetanus in high-income settings disproportionately affects infants born to immigrant or undocumented mothers, a trajectory that reflects a failure of prevention upstream— specifically in primary immunization, antenatal tetanus-containing vaccination, and access to clean delivery services. While migrant-inclusive immunization is a global public health standard, significant implementation gaps persist for families facing linguistic, financial, and legal barriers to routine care. These barriers have been described as structural, involving “entitlement” (legal eligibility), “reachability” (physical and administrative access), and “adherence” (trust and acceptance), all of which must align to ensure coverage (39). Surveillance data from high-income countries underscore this pattern: while tetanus has become rare overall, residual risk clusters heavily in populations with incomplete vaccination histories and limited prenatal engagement. Historical surveillance established that tetanus mortality disproportionately affected the unvaccinated, a finding reinforced by contemporary analyses of vaccine preventable disease burdens in at-risk adults (40, 41). This demographic profile increasingly overlaps with migrant communities, where inconsistent coverage creates pockets of susceptibility (7). Serologic and coverage studies repeatedly document profound immunity gaps among migrant communities compared with native populations, translating into continued vulnerability for pregnant women and their newborns. Recent seroprevalence data from Spain confirm significant susceptibility to VPDs in migrant shelters and referral units (42, 43). These findings are corroborated by a broader systematic review, which identified that pooled immunity coverage for key diseases among migrants in Europe frequently falls below herd immunity thresholds (44). Similarly, it has been highlighted that despite policies promoting catch-up immunization, migrants face higher incidence and mortality for VPDs because of these persistent coverage disparities (45). Furthermore, refugee health assessments consistently demonstrate under immunization at entry, reinforcing the need for systematic catch-up vaccination at the first point of health contact. However, it has been noted that while national regulations often support migrant vaccination, the operating procedures to guarantee access at the community level are frequently fragmented or absent (46). These observations align with the paradox observed in our cohort: modern PICU care can achieve survival for most affected infants, yet the accompanying resource utilization represents the downstream cost of a preventable upstream failure. The specific health vulnerabilities of migrants require evidence-based policy adjustments that transcend acute care. Therefore, prevention must be designed to reach undocumented and migrant families before birth, addressing the “prevention vacuum” rather than merely relying on critical care to rescue infants after disease onset (39, 45).

### Neurodevelopmental sequelae

In our cohort, survival was not synonymous with intact recovery. A total of 14% of survivors screened positive for developmental delay at early follow-up, predominantly among infants who experienced convulsions and prolonged PICU exposure. This observation aligns with historical data indicating that while many survivors recover, severe tetanus associated with frequent spasms and apnea can result in significant morbidity, including cerebral palsy and cognitive deficits, likely attributable to cerebral anoxia (47). Subsequently, it was confirmed that disease severity directly correlates with long-term outcomes; survivors with severe tetanus (Ablett grade ≥ 3) demonstrated significantly lower cognitive domain scores compared with those with mild disease, effectively challenging the older assumption that tetanus survivors invariably escape neurological sequelae (48). Our observation that convulsions associate with later delay is consistent with the broader neonatal seizure literature. Neonatal seizures are not merely symptomatic but are potential contributors to adverse outcomes; notably, status epilepticus serves as a strong independent predictor of subsequent epilepsy and neurodevelopmental impairment (49). Furthermore, while mortality from neonatal seizures has decreased, the prevalence of neurodevelopmental sequelae remains stable at approximately 46%, emphasizing the need to consider seizure burden and underlying etiology when prognosticating (50). Moreover, longer PICU exposure—an aggregate proxy for illness severity, iatrogenic risk, and prolonged analgesia/sedation—must be interpreted within the framework of Post-Intensive Care Syndrome in pediatrics (PICS-p). PICS-p is conceptualized as a constellation of physical, cognitive, and emotional impairments that persist after discharge, necessitating a shift in focus from mere survival to survivorship quality (24). This is corroborated by findings that cognitive and social domains are frequently compromised in pediatric survivors, requiring broad, multidimensional assessment strategies beyond simple mortality statistics (51). These findings support two care imperatives. First, neonatal tetanus survivorship should include structured neurodevelopmental surveillance. Because functional impairments in PICU survivors can be persistent and heterogeneous, standardized screening tools like the Vineland Adaptive Behavior Scales or PedsQL are essential to quantify physical and psychosocial deficits early (52). Second, screening must be coupled with timely early-intervention referral. For undocumented families, follow-up pathways must be designed to be low-barrier and culturally safe. Children in immigrant families often face systemic inequities that threaten their health and wellbeing; consequently, pediatricians must adopt a framework of cultural humility and advocate for policies that delink health access from immigration status to mitigate the amplification of disparities (53). Structured neurodevelopmental follow-up is desirable where feasible; however, survivorship outcomes should be interpreted in the context of postdischarge mediators, including access to rehabilitation, caregiver resources, language and documentation barriers, and continuity of primary care. Because these factors may vary substantially across populations and health systems, future follow-up studies should capture them explicitly before comparing outcomes across settings.

### Prevention and policy implications

Closing upstream gaps: Because neonatal tetanus is almost entirely preventable, the most effective “resource utilization intervention” is positioned upstream: ensuring tetanus-containing vaccination before and during pregnancy, improving antenatal care engagement, and guaranteeing clean delivery and cord-care practices. Global guidance emphasizes that protecting displaced and undocumented populations requires the systematic removal of access barriers. As noted in the UCL–Lancet Commission on Migration and Health, restrictive health policies are often driven by political ideology rather than evidence, whereas inclusive healthcare represents a rational health-system efficiency strategy that prevents high-cost emergency interventions (54). Within Saudi Arabia, maternal vaccination represents a critical actionable lever; however, local evidence indicates that provider-level knowledge gaps and inconsistent counseling remain significant hurdles. Surveys indicate that fewer than half of obstetricians in the Kingdom considered maternal immunization a core responsibility, while patient awareness remains similarly low (55, 56). Furthermore, even when policy shifts occur, such as the landmark 2020 health amnesty during the COVID-19 pandemic, a “trust gap” often persists. An analysis of this amnesty suggests that while the state provided a pardon for undocumented individuals to access care, deep-seated institutional distrust and the fear of future legal repercussions led many to avoid health services, highlighting the need for community-embedded outreach (57). Ultimately, prevention strategies for undocumented migrants must operationalize four elements: (i) the removal of administrative and financial barriers to antenatal care; (ii) the implementation of proactive catch-up vaccination at first contact; (iii) the deployment of culturally and linguistically concordant community outreach to build trust; and (iv) the maintenance of a rigorous “firewall” between clinical services and immigration enforcement. The importance of this firewall is demonstrated by data from inclusive local systems that show that providing a “medical home” and clear legal protections can effectively buffer against the “chilling effect” of enforcement, ensuring that vulnerable families continue to utilize essential preventive services (58, 59). Our findings quantify the downstream stakes: when prevention fails, modern PICU care can deliver survival but at a substantial resource cost and with measurable survivorship morbidity. Closing upstream gaps by aligning Saudi health policies with the universal population health goals of Vision 2030 is therefore both an ethical necessity and a strategic health-system requirement.

## Strengths and limitations

This study provides one of the largest contemporary tertiary-PICU descriptions of neonatal tetanus among undocumented migrant families in Saudi Arabia, using a 10-year cohort, WHO-based case definition, structured chart abstraction, and clinically relevant outcomes such as mortality, invasive ventilation duration, PICU/hospital length of stay, and early developmental screening. The analysis was also strengthened by admission-only prognostic modeling, rare-event methods, and a competing-risk sensitivity analysis to support interpretation of duration-based outcomes.

However, this was a single-center tertiary PICU cohort; therefore, case mix, referral patterns, clinical protocols, and access barriers may differ from other regions and healthcare systems. The findings should be interpreted as a detailed benchmark of one high-volume referral experience rather than a nationally representative estimate. Misclassification of undocumented migrant status is possible because classification relied on retrospective administrative and social-work documentation rather than prospective legal-status verification. To reduce this risk, we used multiple administrative sources and investigator adjudication; nevertheless, residual misclassification cannot be excluded. The small number of deaths limited statistical power and precision; therefore, mortality models should be interpreted as exploratory and hypothesis-generating rather than definitive. The near-uniform Ablett severity distribution also limited robust assessment of severity-grade effects. Developmental findings represent early screening outcomes at 3–6 months among survivors with available follow-up and should not be interpreted as definitive long-term neurodevelopmental diagnoses. Finally, residual confounding remains possible because of the retrospective design. Future work should prioritize harmonized case definitions, transparent treatment documentation, and standardized follow-up measures so that findings from different settings can be compared cautiously rather than pooled without regard to clinical and population differences.

## Future direction

Future work should prioritize harmonized case definitions, transparent documentation of treatment protocols, and standardized follow-up measures so that findings from different settings can be compared cautiously and interpreted in context. Structured neurodevelopmental follow-up is desirable where feasible; however, survivorship outcomes should be evaluated alongside postdischarge mediators, including rehabilitation access, caregiver resources, language and documentation barriers, and continuity of primary care. Public health and implementation studies should also evaluate vaccination access, antenatal engagement, and safe-delivery interventions for undocumented migrant mothers, because elimination of neonatal tetanus in high-income settings ultimately depends less on ICU innovation than on equitable prevention.

## Conclusion

In this 10-year tertiary PICU cohort of neonatal tetanus among undocumented migrant families, in-hospital mortality was low, but survivors required prolonged mechanical ventilation and extended PICU/hospital stays. Because mortality was rare, admission variables should be interpreted as exploratory prognostic signals rather than definitive independent predictors. Shorter observed ventilation or length-of-stay outcomes in clinically unstable infants may reflect death-related truncation, as supported by the competing-risk sensitivity analysis. These findings highlight the substantial critical-care burden and early survivorship morbidity associated with a preventable disease and reinforce the importance of upstream prevention through accessible antenatal care, maternal vaccination, and safe delivery practices.

## Data Availability

The original contributions presented in the study are included in the article/Supplementary Material, further inquiries can be directed to the corresponding author.
