# Supplementary material for: Neonatal tetanus in undocumented migrant families in Saudi Arabia: outcomes and critical care resource utilization in a 10-year tertiary PICU cohort
Source: Front Pediatr. 2026 Aug 19;14:1807654. doi: 10.3389/fped.2026.1807654 (PMC13534090; doi:10.3389/fped.2026.1807654)
Supplement: Supplementary file 1 [file Supplementaryfile1.pdf]

Supplementary Documents

Supplementary Table S1. Expanded baseline characteristics and admission features (overall cohort; N = 74).

Characteristic	Overall (N=74)
**Sociodemographic descriptors**	
**Nationality, n (%)**	
Chad	22 (29.7)
Somalia	21 (28.4)
Myanmar	19 (25.7)
Pakistan	5 (6.8)
Others	7 (9.5)
**Time in country (registration category), n (%)**	
> 10 years	24 (32.4)
1–5 years	19 (25.7)
5–10 years	17 (23.0)
Unknown	12 (16.2)
< 1 year	2 (2.7)
**Delivery and cord-care characteristics (expanded)**	
**Place of delivery, n (%)**	
Home	65 (87.8)
Polyclinic	6 (8.1)
Car	3 (4.1)
**Cord cutting practice, n (%)**	
Uncleaned	47 (63.5)
Cleaned	27 (36.5)
Dirty cord on presentation, n (%)	48 (64.9)
**Admission clinical signs (expanded), n (%)**	
Inability to suck (at presentation), n (%)	74 (100.0)
Jerkings/spasms (as recorded), n (%)	74 (100.0)
Trismus/lockjaw, n (%)	68 (91.9)
Opisthotonus/arching, n (%)	24 (32.4)

Characteristic	Overall (N=74)
Convulsions/spasms, n (%)	31 (41.9)
Fever, n (%)	64 (86.5)

Footnote: This table provides additional cohort descriptors not shown in Table 1 (main text), including nationality distribution, time in country (administrative registration category), expanded delivery and cord-care characteristics, and additional admission clinical signs. Data are presented as n (%), with percentages calculated using N = 74. "Others" includes all remaining nationalities (including Yemen) not listed separately.

Table S2: Rare-events ridge logistic regression (intercept unpenalized) modeling in-hospital mortality using baseline covariates only.

Predictor	Coefficient (ridge, log-odds)	Shrunken OR	95% CI (OR scale)
Ablett III+ (vs I–II)	0.09	1.10	0.17–7.22
Age (per SD)	−0.71	0.49	0.15–1.56
Weight (per SD)	−0.97	0.38	0.12–1.23
Convulsions present	0.99	2.69	0.55–13.19
Lockjaw/trismus present	0.13	1.14	0.18–7.35
CRP (per SD)	0.62	1.86	0.69–5.01
CK/CPK (per SD)	−0.55	0.58	0.14–2.31
Male sex	0.02	1.02	0.21–4.90

Notes: ORs are penalization-shrunk estimates from ridge rare-events logistic regression (intercept unpenalized). Continuous predictors were standardized to cohort SD; categorical predictors are coded present vs absent. Reported are penalization-shrunk odds ratios (OR) with Wald 95% confidence intervals (CI). Given the small number of deaths (n=3), results emphasize direction and magnitude with appropriate caution; no post-admission treatments were included to avoid confounding by indication.

Table S3: Rare-events ridge logistic regression (intercept penalized) for in-hospital mortality (baseline-only)

Predictor	Coefficient (ridge, log-odds)	Shrunken OR	95% CI (profile likelihood, OR scale)
Ablett III+ (vs I–II)	0.09	1.10	0.17–7.66
Convulsion present	0.99	2.69	0.55–14.36
CRP (per SD)	0.62	1.86	0.65–5.37
Age at onset (per SD)	−0.71	0.49	0.13–1.41
Weight (per SD)	−0.97	0.38	0.10–1.19

Notes: Penalized ridge rare-events logistic regression with the intercept penalized. Continuous predictors were standardized to the cohort SD; binary predictors coded present vs absent. Odds ratios (OR) are penalization-shrunk;

confidence intervals are profile-likelihood 95% CIs. With only three deaths in the cohort, estimates are imprecise and should be interpreted as hypothesis-generating.

Table S4: Bayesian logistic regression for in-hospital mortality using weakly informative priors (baseline-only predictors).

Parameter	Posterior median OR	95% credible interval (CrI)
Ablett grade III+ (vs I–II)	2.94	0.15–56.24
Birth weight (per SD)	0.24	0.04–1.29
Age at onset (per SD)	0.22	0.03–1.45
CRP (per SD)	2.12	0.65–6.96
Convulsions present (yes vs no)	12.47	0.54–286.66

Footnotes: Bayesian logistic regression used weakly informative priors to stabilize estimation under rare events (3 deaths). Continuous predictors were z-standardized; odds ratios are expressed per 1 SD increase. Posterior summaries are reported as median OR with approximate 95% credible intervals (CrI). Predictors were restricted to baseline/admission variables (post-admission therapies excluded to reduce confounding by indication). Abbreviations: CrI, credible interval; CRP, C-reactive protein; OR, odds ratio; SD, standard deviation.

Table S5: Sensitivity mortality models using Firth bias-reduced logistic regression (baseline-only predictors).

Model specification	Predictor	Adjusted OR	95% CI
Primary (Firth)	Ablett grade III+ (vs lower/unknown)	12.02	0.58–250.60
Limited (Firth)	Ablett grade III+ (vs lower/unknown)	11.00	0.52–231.74
Limited (Firth)	Delivery setting (home vs facility)	NE†	—

Footnotes: Firth penalized-likelihood logistic regression was used to mitigate small-sample bias and separation. The primary model included bedside severity (Ablett grade III+ vs lower/unknown). The limited specification additionally included delivery setting. Adjusted odds ratios (aOR) are shown with profile-likelihood 95% confidence intervals. **NE†** indicates not estimable due to quasi-separation/near-constant exposure (delivery overwhelmingly occurred at

home), yielding unstable or undefined coefficients. Cohort size was N = 74 with 3 deaths; estimates are imprecise and should be interpreted as sensitivity analyses

Table S6. Neurodevelopmental screening status at 3–6 months among survivors and associated ICU resource utilization (n = 71).

Variable	Screen positive (n=10)	Age-appropriate (n=61)	P value
Convulsions during admission — n (%)	8 (80.0%)	20 (32.8%)	0.011
PICU length of stay, days — median (IQR)	36.5 (26.8–59.0)	30.0 (18.8–36.2)	0.035
IMV duration, days — median (IQR)	28.5 (18.8–38.8)	23.0 (15.2–29.8)	0.245
Hospital length of stay, days — median (IQR)	54.0 (36.0–72.0)	40.0 (26.0–56.0)	0.056
Admission CRP, mg/L — median (IQR)	56.9 (27.8–120.0)	25.7 (10.8–67.0)	0.153

Footnote: Continuous variables are presented as median (IQR) and compared using the Mann–Whitney U test; categorical variables are presented as n (%) and compared using Fisher's exact test. Abbreviations: CRP, C-reactive protein; IMV, invasive mechanical ventilation; IQR, interquartile range; PICU, pediatric intensive care unit.

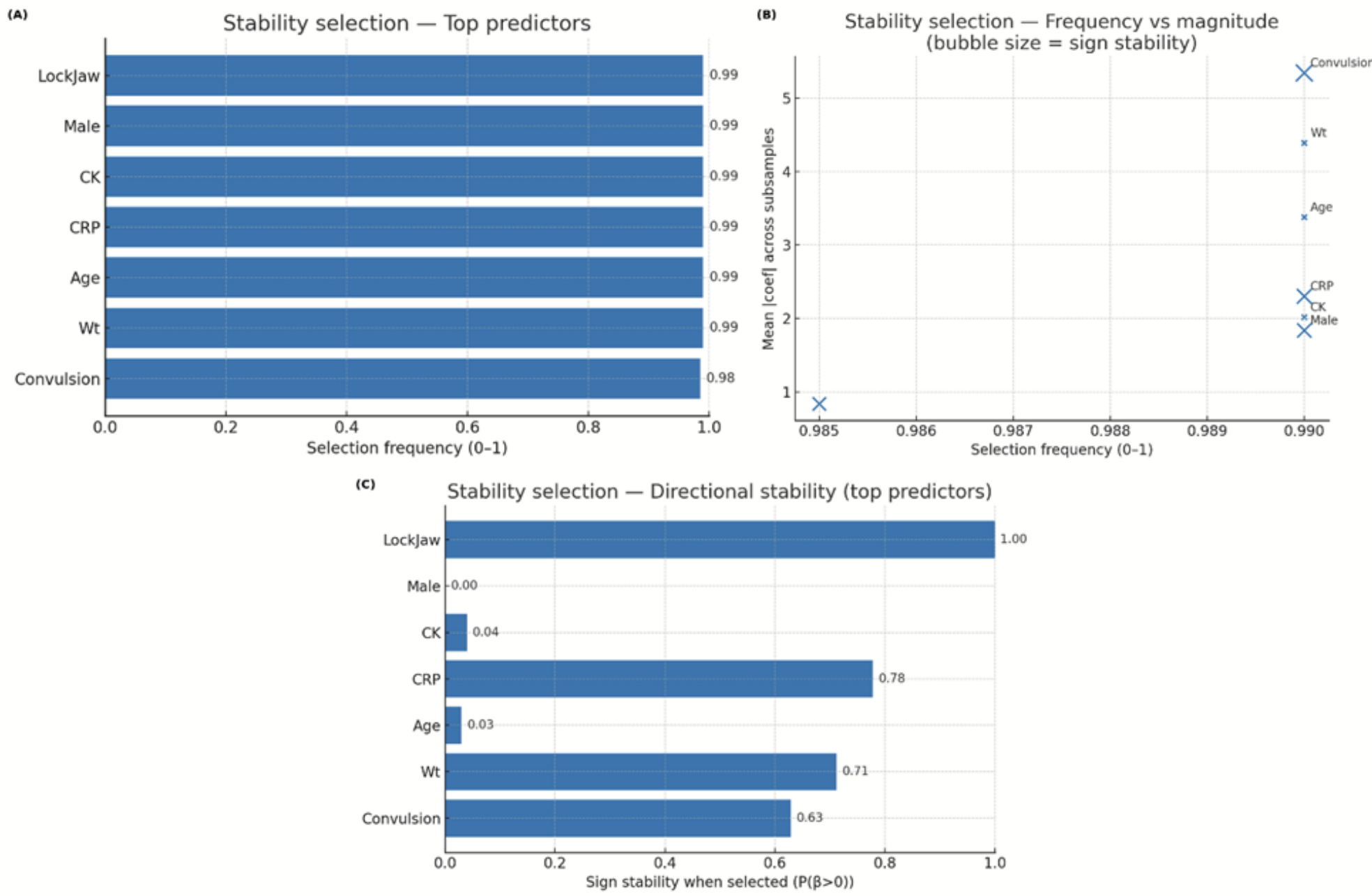

Figure S1 . Elastic-net stability selection for the composite severe outcome: Class-weighted elastic-net logistic regression with stability selection (200 subsamples; slopes penalized; intercept unpenalized) was applied to admission covariates only. Continuous variables were z-standardized, and post-admission therapies were excluded. The composite outcome was defined as death OR septic shock OR IMV ≥14 days OR VAP/CLABSI.**(A)** Selection frequency (0–1) for the top predictors; higher values indicate greater robustness across subsamples.**(B)** Selection frequency versus average coefficient magnitude; bubble size encodes directional stability.**(C)** Directional stability (P[β>0]) for the top predictors; values near 1.0 indicate consistently risk-increasing direction, whereas values near 0 indicate consistently protective direction. These findings are exploratory and hypothesis-generating and should not be interpreted as mortality-specific prediction.

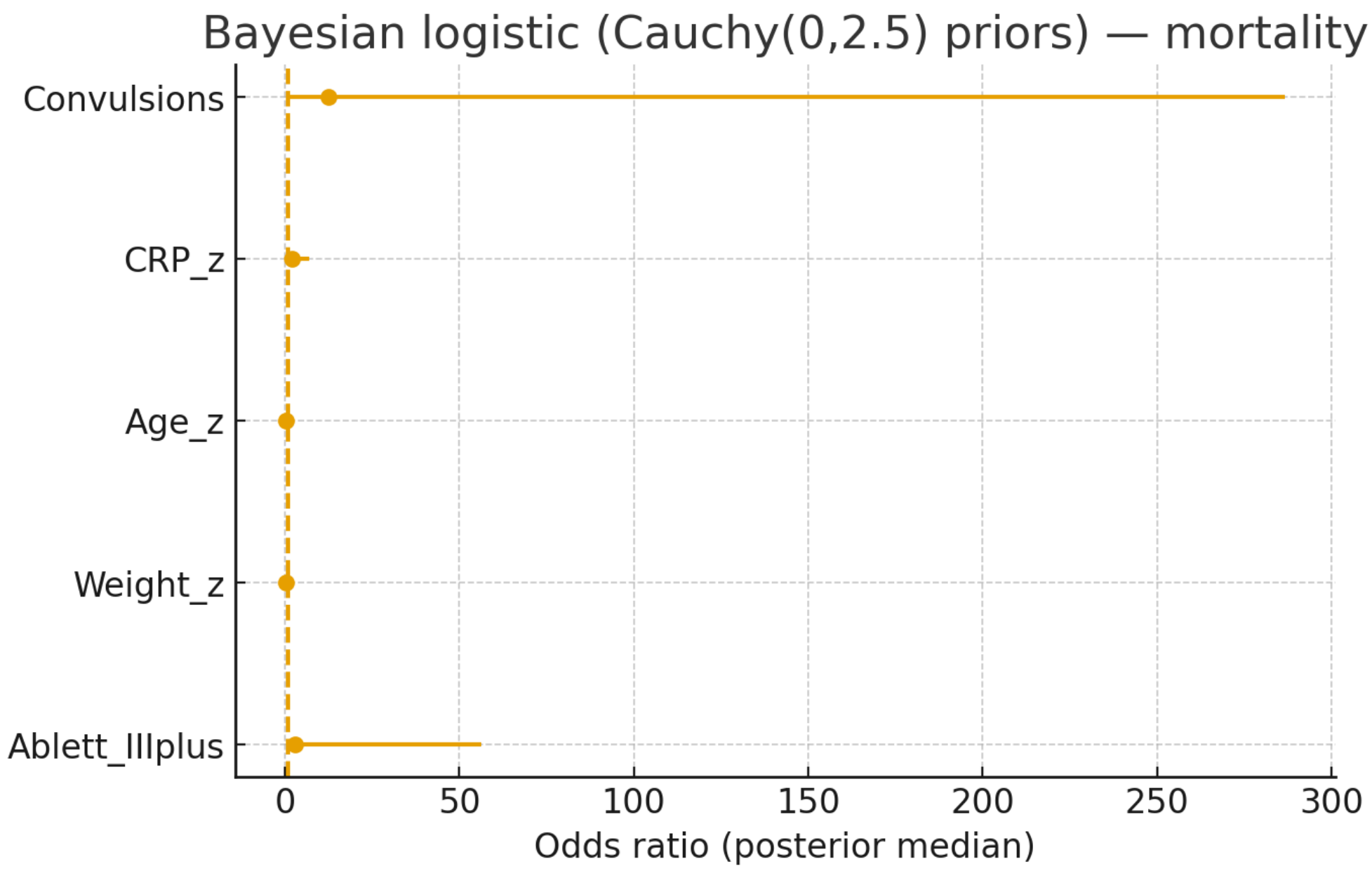

Figure S2. Bayesian logistic regression for in-hospital mortality (posterior medians and 95% credible intervals). Forest plot of odds ratios from a Bayesian logistic model fit to baseline variables only (N=74; deaths=3). Points show posterior median ORs and horizontal bars the ~95% credible intervals (Laplace/MAP approximation). A vertical dashed line marks OR=1 (no association). Slopes used weakly-informative Cauchy (0,2.5) priors; the intercept had a weak normal prior. Continuous predictors (Weight, Age_z, CRP_z) were standardized per cohort SD; binary predictors (Ablett III+, Convulsions) were coded 0/1. Values >1 indicate higher odds of death; <1 indicate lower odds. Intervals are wide because only three deaths occurred; results are hypothesis-generating.

Supplementary Figure. Competing-risk sensitivity analysis using cumulative incidence functions

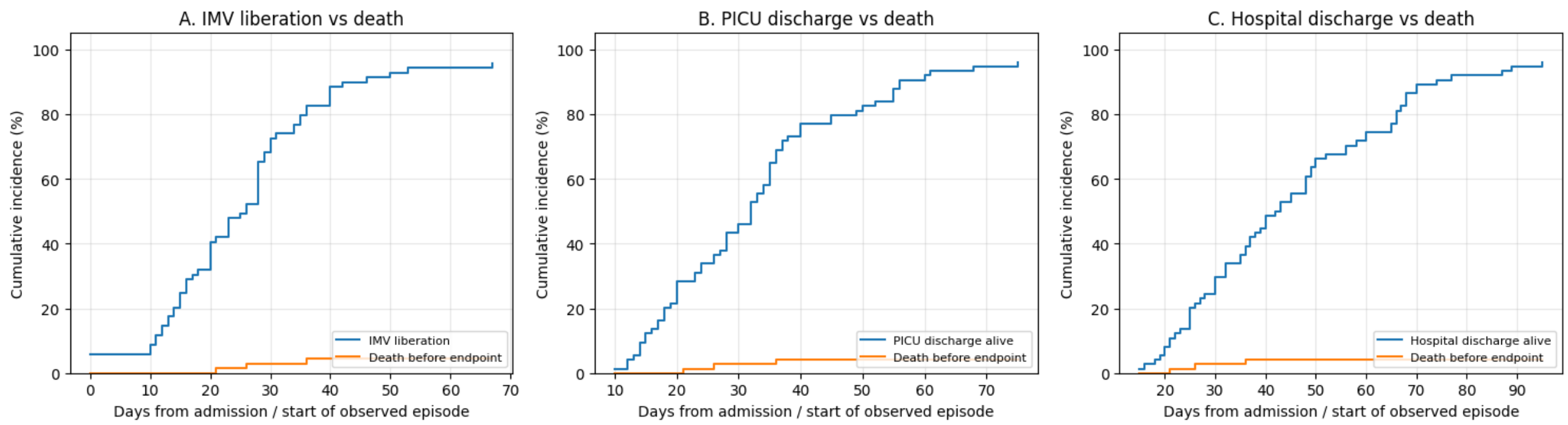

Figure S3. Competing-risk sensitivity analysis for invasive mechanical ventilation (IMV) liberation, PICU discharge, and hospital discharge among neonates with tetanus. Cumulative incidence functions (CIFs) are shown for (A) successful liberation from invasive mechanical ventilation, (B) discharge alive from the pediatric intensive care unit (PICU), and (C) discharge alive from hospital, with death treated as a competing event. The blue curves represent the cumulative incidence of the outcome of interest, whereas the orange curves represent the cumulative incidence of death before the corresponding outcome occurred. Across all endpoints, the cumulative incidence of death remained low (3 deaths; 4.1% overall mortality), while the majority of infants ultimately achieved extubation, PICU discharge, and hospital discharge. These findings support the interpretation of the primary accelerated failure-time (AFT) analyses by demonstrating that shorter observed durations among clinically unstable infants may reflect mortality-related truncation of follow-up rather than accelerated clinical recovery. Because only three deaths occurred in the cohort, the competing-risk analysis is presented as a descriptive sensitivity analysis and was not used for formal regression modeling. Abbreviations: CIF, cumulative incidence function; IMV, invasive mechanical ventilation; PICU, pediatric intensive care unit.
